# Clinicopathological features, surgical strategy and prognosis of duodenal gastrointestinal stromal tumors: a series of 300 patients

**DOI:** 10.1186/s12885-018-4485-4

**Published:** 2018-05-15

**Authors:** Zhen Liu, Gaozan Zheng, Jinqiang Liu, Shushang Liu, Guanghui Xu, Qiao Wang, Man Guo, Xiao Lian, Hongwei Zhang, Fan Feng

**Affiliations:** 1 Division of Digestive Surgery, Xijing Hospital of Digestive Diseases, the Fourth Military Medical University, 127 West Changle Road, 710032, Xi’an, Shaanxi Province China; 2 Department of General Surgery, No.1 Hospital of PLA, 74 Jingning Road, Lanzhou, 730030 China; 3 Cadre’ s sanitarium, 62101 Army of PLA, 67 Nahu Road, Xinyang, 464000 Henan China; 4 Department of General Surgery, No. 91 Hospital of PLA, 239 Gongye Road, Jiaozuo, 454000 Henan China

**Keywords:** Duodenum, Gastrointestinal stromal tumor, Features, Surgery, Prognosis

## Abstract

**Background:**

The relatively low incidence of duodenal gastrointestinal stromal tumors (GISTs) and the unique anatomy make the surgical management and outcomes of this kind of tumor still under debate. Thus, this study aimed to explore the optimal surgical strategy and prognosis of duodenal GISTs.

**Methods:**

A total of 300 cases of duodenal GISTs were obtained from our center (37 cases) and from case reports or series (263 cases) extracted from MEDLINE. Clinicopathological features, type of resections and survivals of duodenal GISTs were analyzed.

**Results:**

The most common location of duodenal GISTs was descending portion (137/266, 51.5%). The median tumor size was 4 cm (0.1–28). Most patients (66.3%) received limited resection (LR). Pancreaticoduodenectomy (PD) was mainly performed for GISTs with larger tumor size or arose from descending portion (both *P* < 0.05). For both the entire cohort and tumors located in the descending portion, PD was not an independent risk factor for disease-free survival (DFS) and disease-specific survival (DSS) (both *P* > 0.05). Duodenal GISTs were significantly different from gastric GISTs with respect to tumor size, mitotic index and NIH risk category (all *P* < 0.05). The DFS and DSS of duodenal GISTs was significantly worse than that of gastric GISTs (both *P* < 0.05).

**Conclusions:**

LR was a more prevalent surgical procedure and PD was mainly performed for tumors with larger diameter or located in descending portion. Type of resection was not an independent risk factor for the prognosis of duodenal GISTs. Prognosis of duodenal GISTs was significantly worse than that of gastric GISTs.

**Electronic supplementary material:**

The online version of this article (10.1186/s12885-018-4485-4) contains supplementary material, which is available to authorized users.

## Background

Gastrointestinal stromal tumor (GIST) is the commonest mesenchymal tumor in alimentary tract representing an annual incidence of 10 cases per million people worldwide [1]. While this kind of tumor could originate from the interstitial Cajal cells (ICC) throughout the entire alimentary tract, GISTs are mostly found in the stomach (60–70%), small intestine (20–30%) and colorectum (10%) [2]. Notably, only 1–5% GISTs occurred in the duodenum [3]. Thus, the research on duodenal GIST was lacking due to its rare incidence.

To date, complete resection without lymph node clearance is the standard curative treatment for primary localized GISTs [4, 5]. However, the optimal surgical procedure for duodenal GISTs is not well defined due to their complex anatomy around the pancreaticoduodenal region [6–8]. The limited resection (LR) is reported to be a technically feasible and oncologically sound procedure for duodenal GISTs, while the pancreaticoduodenectomy (PD) is also warranted in some cases due to the anatomical considerations of the proximity of critical structures, including the papilla, pancreas and biliary and pancreatic ducts [9–12]. However, the survival impact of surgical procedure on duodenal GISTs still remains controversial [6, 13, 14].

Thus, the current study aimed to investigate the optimal surgical strategy and prognosis of duodenal GISTs based on the largest sample size so far.

## Methods

Thirty-seven cases of duodenal GISTs which were diagnosed and treated in our center from May 2010 to November 2016, and 263 cases of duodenal GISTs reported in the literature were enrolled into this study. Literature published in English from 1st January 2000 to 1st January 2017 were searched in the database of MEDLINE using the following keywords: (GIST OR gastrointestinal stromal tumor OR gastrointestinal stromal tumour OR GISTs OR gastrointestinal stromal tumors OR gastrointestinal stromal tumours OR extragastrointestinal stromal tumor OR extragastrointestinal stromal tumors OR extragastrointestinal stromal tumour OR extragastrointestinal stromal tumours) AND (duodenum OR duodenal). The research resulted in 101 eligible case reports or series [8, 10, 12, 15–112] including 263 cases of duodenal GISTs. Finally, a total of 300 cases of duodenal GISTs were identified in our study (Additional file 1). In addition, the clinicopathological features and prognosis of duodenal GISTs were compared with 378 gastric GISTs which were diagnosed and treated from May 2010 to November 2016 in our center. This study was approved by the Ethics Committee of Xijing Hospital, and written informed consents were obtained from the patients.

Clinicopathological factors including age, gender, preoperative symptoms, anatomical location, surgical procedure, resection margin, tumor size, mitotic index, morphology, immunohistochemistry, genomic mutation, National Institutes of Health risk category (NIH), adjuvant therapy and survival data were collected. The GISTs were classified as very low, low, intermediate and high risk following the modified protocol of NIH risk classification reported by Joensuu [113].

For survival analysis, the exclusion criteria were listed as follows (Both for duodenal and gastric GISTs): 1) accompanied with other malignant tumors or GISTs in other locations; 2) with distant metastasis or tumor rupture; 3) with neoadjuvant therapy; 4) not received R0 resection; 5) without follow-up records. Because of data acquisition, completeness of data is limited. Finally, a total of 202 patients of duodenal GISTs and 253 patients of gastric GISTs were included for survival analysis.

Data were processed using SPSS 22.0 for Windows (SPSS Inc., Chicago, IL). Numerical variables were expressed as the mean ± SD unless otherwise stated. Discrete variables were analyzed using the Chi-square test or Fisher’s exact test. Risk factors for survival were identified by univariate analysis and Cox proportional hazards regression model was used for multivariate analysis. Evaluation for disease-free survival (DFS) and disease-specific survival (DSS) were obtained by the Kaplan-Meier method and differences between curves were compared using log-rank test. The *P*-values were considered to be statistically significant at the 5% level.

## Results

The clinicopathological characteristics of 300 duodenal GISTs were summarized in Table 1. There were 143 male (49.1%) and 148 female (50.8%). The patient age ranged from 7 to 84 years (mean, 56 years; median, 57 years). The most common symptom was bleeding (128/300, 42.7%) followed by abdominal pain (56/300, 18.7%). Descending portion was the most common site (137/266, 51.5%), followed by horizontal portion (65/266, 24.4%), superior portion (42/266, 15.8%) and ascending portion (22/266, 8.3%). R0 resection was performed for the 91.7% of the patients. There were only 2 patients that underwent R1 or R2 resection. One hundred and ninety-nine (66.3%) patients received LR and 78 (26.0%) patients received PD. The tumors ranged from 0.1 cm to 28 cm (mean: 5.6 cm; median: 4 cm) in maximum diameter. The mitotic index of 59 (24.6%) patients exceeded 5/50 high-power field (HPF). One hundred and twenty-seven patients (49.2%) were classified as high risk by the NIH risk category, and 104 patients (40.3%) were at low risk. A total of 13 (4.3%) patients received neoadjuvant therapy and 37 (12.3%) patients received imatinib therapy after surgery.

**Table 1:** Clinicopathological characteristics of 300 cases of duodenal GISTs

Characteristics	Parameters
Age (∑ = 267)	
≤ 60	161 (60.3%)
> 60	106 (39.7%)
Gender (∑ = 291)	
Male	143 (49.1%)
Female	148 (50.8%)
Symptoms (∑ = 300)	
Bleeding or anemia	128 (42.7%)
Abdominal pain	56 (18.7%)
Abdominal mass	11 (3.7%)
Abdominal discomfort	11 (3.7%)
Anorexia	6 (2.0%)
Othersa^a^	36 (12.0%)
Anatomical location (∑ = 266)	
Superior portion	42 (15.8%)
Descending portion	137 (51.5%)
Horizontal portion	65 (24.4%)
Ascending portion	22 (8.3%)
Surgical procedure (∑ = 300)	
Limited resection*	199 (66.3%)
Pancreaticoduodenectomy	78 (26.0%)
Not available	13 (4.3%)
No surgery	10 (3.3%)
Resection margin (∑ = 300)	
R0	275 (91.7%)
R1/2	2 (0.7%)
Not available/No surgery	23 (7.7%)
Tumor size (∑ = 277)	
≤ 2 cm	34 (12.3%)
2–5 cm	135 (48.7%)
5–10 cm	73 (26.4%)
> 10 cm	35 (12.6%)
Mitotic index (∑ = 240)	
≤ 5	181 (75.4%)
> 5	59 (24.6%)
Morphology (∑ = 160)	
Spindle	148 (92.5%)
Epithelioid	1 (0.6%)
Mixed	11 (6.9%)
Immunohistochemistry	
CD117 (∑ = 288)	284 (98.6%)
DOG-1 (∑ = 41)	40 (97.6%)
CD34 (∑ = 167)	126 (75.4%)
Genomic mutation (∑ = 41)	
KIT	31 (75.6%)
PDGFRA	1 (2.4%)
KIT and PDGFRA	5 (12.2%)
Wild type	4 (9.8%)
NIH risk category (∑ = 258)	
Very low	25 (9.7%)
Low	104 (40.3%)
Intermediate	2 (0.8%)
High	127 (49.2%)
Neoadjuvant therapy (∑ = 300)	
No	287 (95.7%)
Yes	13 (4.3%)
Adjuvant therapy (∑ = 300)	
No	263 (87.7%)
Yes	37 (12.3%)
Follow-up (∑ = 202, month)	
Mean ± SD	39.3 ± 39.4
Median (range)	25.0 (13.0, 58.5)
Survival rate (∑ = 202)	
1−/3−/5−/10-year DFS	94.4%/75.2%/64.4%/46.5%
1−/3−/5−/10-year DSS	99.5%/93.4%/80.9%/54.5%

^a^Limited resection included wedge resection, segmental resection or enucleation

GIST: gastrointestinal stromal tumor; NIH: National Institutes of Health; SD: standard deviation

**Fig. 1:**
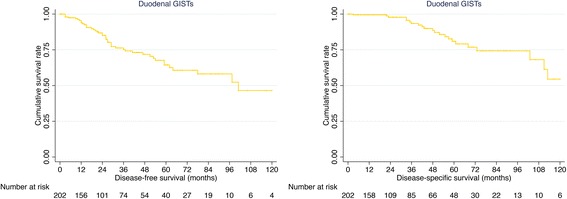
DFS and DSS of duodenal GISTs

Survival data of 202 patients with duodenal GISTs were eventually selected for analysis using exclusion criteria described in the methods section (Table 1). The median follow-up time was 25.0 months (mean: 39.3 months). As shown in Fig. 1, the 1−/3−/5−/10-year DFS of duodenal GISTs was 94.4, 75.2, 64.4 and 46.5%, respectively. The 1−/3−/5−/10-year DSS was 99.5, 93.4, 80.9 and 54.5%, respectively.

**Fig. 2:**
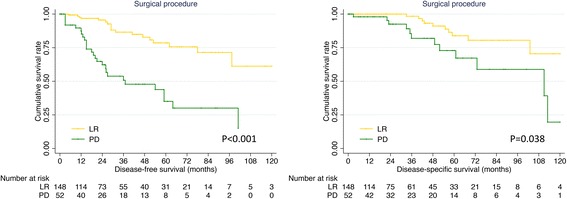
DFS and DSS of duodenal GISTs stratified by surgical procedure

The clinicopathological characteristics of duodenal GISTs received different surgical procedures were compared in Table 2, the tumors underwent PD were mainly located in descending portion (52/77, 88.1%), and had larger diameter, higher mitotic index and higher NIH risk category (all *P* < 0.001). Prognostic factors for duodenal GISTs according to univariate and multivariate analysis were summarized in Table 3. Surgical procedure, tumor size, mitotic index and NIH risk category were risk factors for both DFS and DSS (all *P* < 0.05). Patients underwent LR had a higher 5-year DFS (78.6% vs 35.1%, *P* < 0.001) and DSS (83.9% vs 72.9%, *P* = 0.008) than patients underwent PD according to Kaplan-Meier analysis (Fig. 2). However, multivariate analysis showed that surgical procedure was not an independent prognostic factor (*P* > 0.05).

**Table 2:** Comparison of clinicopathological factors of duodenal GISTs according to surgical procedure

Characteristics	Limited resection (*n* = 200)	Pancreaticoduodenectomy (*n* = 77)	*P* value
Age			0.782
≤ 60	101 (60.5%)	48 (62.3%)	
> 60	66 (39.5%)	29 (37.7%)	
Gender			0.205
Male	100 (51.3%)	32 (42.7%)	
Female	95 (48.7%)	43 (57.3%)	
Anatomical location			< 0.001
Superior portion	37 (19.7%)	3 (5.1%)	
Descending portion	73 (38.8%)	52 (88.1%)	
Horizontal portion	56 (29.8%)	4 (6.8%)	
Ascending portion	22 (11.7%)	0	
Tumor size			< 0.001
≤ 2 cm	28 (15.1%)	3 (4.1%)	
2–5 cm	102 (54.8%)	25 (33.8%)	
5–10 cm	37 (19.9%)	32 (43.2%)	
> 10 cm	19 (10.2%)	14 (18.9%)	
Mitotic index			< 0.001
≤ 5	144 (83.2%)	32 (52.5%)	
> 5	29 (16.8%)	29 (47.5%)	
Morphology			0.146
Spindle	100 (94.3%)	39 (86.7%)	
Epithelioid	0	1 (2.2%)	
Mixed	6 (5.7%)	5 (11.1%)	
NIH risk category			< 0.001
Very low	23 (12.8%)	2 (2.8%)	
Low	87 (48.6%)	15 (21.1%)	
Intermediate	2 (1.1%)	0	
High	67 (37.4%)	54 (76.1%)	
Neoadjuvant therapy			0.472
No	194 (97.0%)	73 (94.8%)	
Yes	6 (3.0%)	4 (5.2%)	
Adjuvant therapy			0.362
No	165 (82.5%)	67 (87.0%)	
Yes	35 (17.5%)	10 (13.0%)	

GIST: gastrointestinal stromal tumor; NIH: National Institutes of Health

**Table 3:** Univariate and multivariate analysis of prognostic factors for duodenal GISTs

Prognostic factors	Univariate analysis			Multivariate analysis		
	β	Hazard ratio (95% CI)	*P* value	β	Hazard ratio (95% CI)	*P* value
DFS						
Age	0.480	1.616 (0.874–2.987)	0.126			
Gender	−0.148	0.862 (0.470–1.582)	0.632			
Anatomical location	0.108	1.114 (0.744–1.666)	0.601			
Surgical procedure	1.517	4.559 (2.510–8.281)	< 0.001			
Tumor size	1.441	4.224 (2.769–6.442)	< 0.001	1.406	4.082 (1.979–8.416)	< 0.001
Mitotic index	2.049	7.759 (3.751–16.048)	< 0.001	1.294	3.648 (1.375–9.680)	0.009
NIH risk category	1.457	4.294 (2.394–7.702)	< 0.001			
Adjuvant therapy	0.327	1.387 (0.514–3.742)	0.518			
DSS						
Age	0.338	1.403 (0.596–3.300)	0.438			
Gender	0.324	1.383 (0.587–3.257)	0.459			
Anatomical location	0.107	1.113 (0.649–1.909)	0.697			
Surgical procedure	1.082	2.952 (1.274–6.837)	0.012			
Tumor size	1.629	5.100 (2.640–9.853)	< 0.001	1.339	3.816 (1.743–8.354)	0.001
Mitotic index	1.719	5.580 (2.277–13.674)	< 0.001			
NIH risk category	1.035	2.815 (1.547–5.123)	0.001			
Adjuvant therapy	−1.388	0.249 (0.033–1.877)	0.178			

GIST: gastrointestinal stromal tumor; NIH: National Institutes of Health;

DFS: disease-free survival; DSS: disease-specific survival; CI: confidence interval

**Fig. 3:**
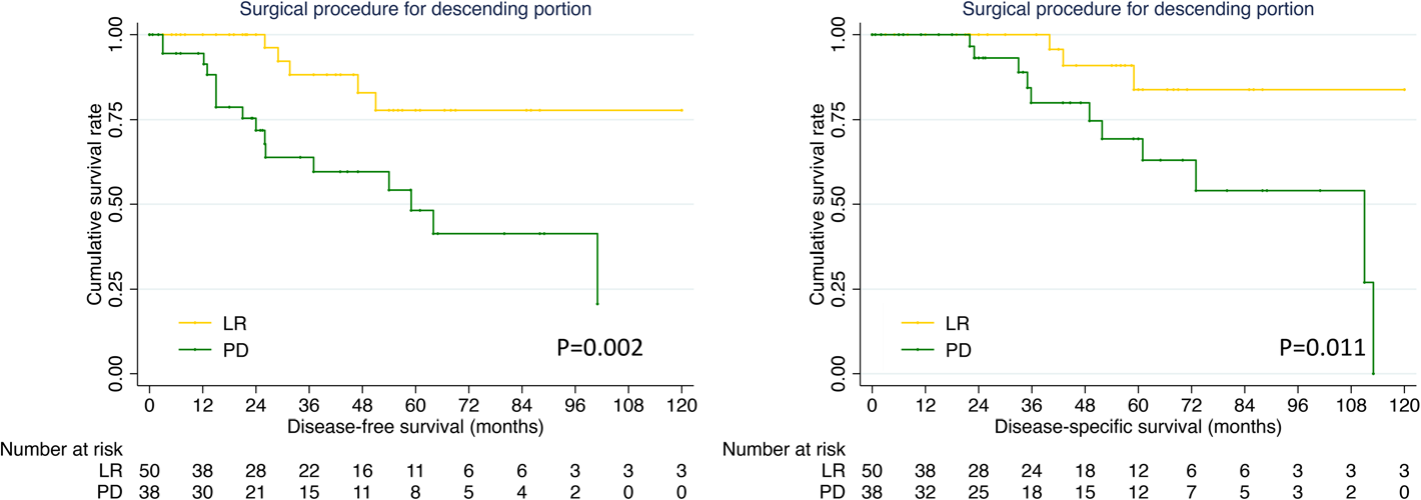
DFS and DSS of descending duodenal GISTs stratified by surgical procedure

Since more than half of duodenal GIST occur at the descending portion, we specifically studied the clinicopathological features of these GISTs based on the type of resection in Table 4. A higher prevalence of large tumor, high mitotic index and high risk category was observed in the descending tumors received PD (all *P* < 0.05). Univariate analysis showed that surgical procedure, tumor size, mitotic index and NIH category were risk factors for both DFS and DSS (Table 5, all *P* < 0.05). As shown in Fig. 3, LR brought a more favorable 5-year DFS (77.8% vs 48.2%, *P* = 0.002) and DSS (83.9% vs 69.3%, *P* = 0.011) than PD. However, multivariate analysis showed that surgical procedure was not an independent prognostic factor (Table 5, *P* > 0.05).

**Table 4:** Comparison of clinicopathological factors of descending duodenal GISTs according to surgical procedure

Characteristics	Limited resection (*n* = 73)	Pancreaticoduodenectomy (*n* = 52)	*P* value
Age			0.160
≤ 60	31 (54.4%)	30 (68.2%)	
> 60	26 (45.6%)	14 (31.8%)	
Gender			0.976
Male	27 (42.9%)	22 (43.1%)	
Female	36 (57.1%)	29 (56.9%)	
Tumor size			0.035
≤ 2 cm	12 (17.4%)	2 (4.0%)	
2–5 cm	33 (47.8%)	19 (38.0%)	
5–10 cm	17 (24.6%)	20 (40.0%)	
> 10 cm	7 (10.1%)	9 (18.0%)	
Mitotic index			< 0.001
≤ 5	50 (86.2%)	25 (54.3%)	
> 5	8 (13.8%)	21 (45.7%)	
Morphology			0.165
Spindle	35 (97.2%)	27 (84.4%)	
Epithelioid	0	1 (3.1%)	
Mixed	1 (2.8%)	4 (12.5%)	
NIH risk category			0.001
Very low	10 (15.4%)	1 (2.1%)	
Low	28 (43.1%)	11 (22.9%)	
Intermediate	0	0	
High	27 (41.5%)	36 (75.0%)	
Neoadjuvant therapy			0.401
No	69 (94.5%)	51 (98.1%)	
Yes	4 (5.5%)	1 (1.9%)	
Adjuvant therapy			0.777
No	33 (80.5%)	30 (83.3%)	
Yes	8 (19.5%)	6 (16.7%)	

GIST: gastrointestinal stromal tumor; NIH: National Institutes of Health

**Table 5:** Univariate and multivariate analysis of prognostic factors for the descending duodenal GISTs

Prognostic factors	Univariate analysis			Multivariate analysis		
	β	Hazard ratio (95% CI)	*P* value	β	Hazard ratio (95% CI)	*P* value
DFS						
Age	0.487	1.628 (0.632–4.193)	0.313			
Gender	−0.375	0.687 (0.279–1.694)	0.415			
Surgical procedure	1.473	4.361 (1.597–11.909)	0.004			
Tumor size	1.445	4.240 (2.073–8.672)	< 0.001	1.721	5.590 (2.144–14.570)	< 0.001
Mitotic index	1.696	5.453 (2.065–14.400)	0.001			
NIH risk category	1.456	4.288 (1.594–11.531)	0.004			
Adjuvant therapy	0.544	1.724 (0.367–8.103)	0.491			
DSS						
Age	0.276	1.318 (0.432–4.019)	0.627			
Gender	0.008	1.008 (0.316–3.218)	0.989			
Surgical procedure	1.519	4.569 (1.260–16.569)	0.021			
Tumor size	2.142	8.515 (2.496–29.053)	0.001	1.976	7.213 (2.138–24.338)	0.001
Mitotic index	1.567	4.792 (1.428–16.087)	0.011			
NIH risk category	1.143	3.136 (1.150–8.552)	0.026			
Adjuvant therapy	−3.181	0.042 (0.000–212.916)	0.465			

GIST: gastrointestinal stromal tumor; NIH: National Institutes of Health;

DFS: disease-free survival; DSS: disease-specific survival; CI: confidence interval

The clinicopathological characteristics of 300 duodenal GISTs including age, gender, tumor size, mitotic index, morphology and NIH risk category were compared with 378 gastric GISTs from out center (Table 6). The tumor size, mitotic index and NIH risk category were significantly different between the two groups (all *P* < 0.001).

**Table 6:** Comparison of clinicopathological characteristics between duodenal and gastric GISTs

Characteristics	Duodenum (*n* = 300)	Stomach (*n* = 378)	*P* value
Age			0.791
≤ 60	161 (60.3%)	224 (59.3%)	
> 60	106 (39.7%)	154 (40.7%)	
Gender			0.826
Male	143 (49.1%)	189 (50.0%)	
Female	148 (50.9%)	189 (50.0%)	
Tumor size			< 0.001
≤ 2 cm	34 (12.3%)	126 (33.5%)	
2–5 cm	135 (48.7%)	138 (36.7%)	
5–10 cm	73 (26.4%)	86 (22.9%)	
> 10 cm	35 (12.6%)	26 (6.9%)	
Mitotic index			< 0.001
≤ 5	181 (75.4%)	225 (61.1%)	
> 5	59 (24.6%)	143 (38.9%)	
Morphology			0.825
Spindle	148 (92.5%)	341 (93.9%)	
Epithelioid	1 (0.6%)	2 (0.6%)	
Mixed	11 (6.9%)	20 (5.5%)	
NIH risk category			< 0.001
Very low	25 (9.7%)	105 (28.5%)	
Low	104 (40.3%)	97 (26.3%)	
Intermediate	2 (0.8%)	87 (23.6%)	
High	127 (49.2%)	80 (21.7%)	

GIST: gastrointestinal stromal tumor; NIH: National Institutes of Health

**Fig. 4:**
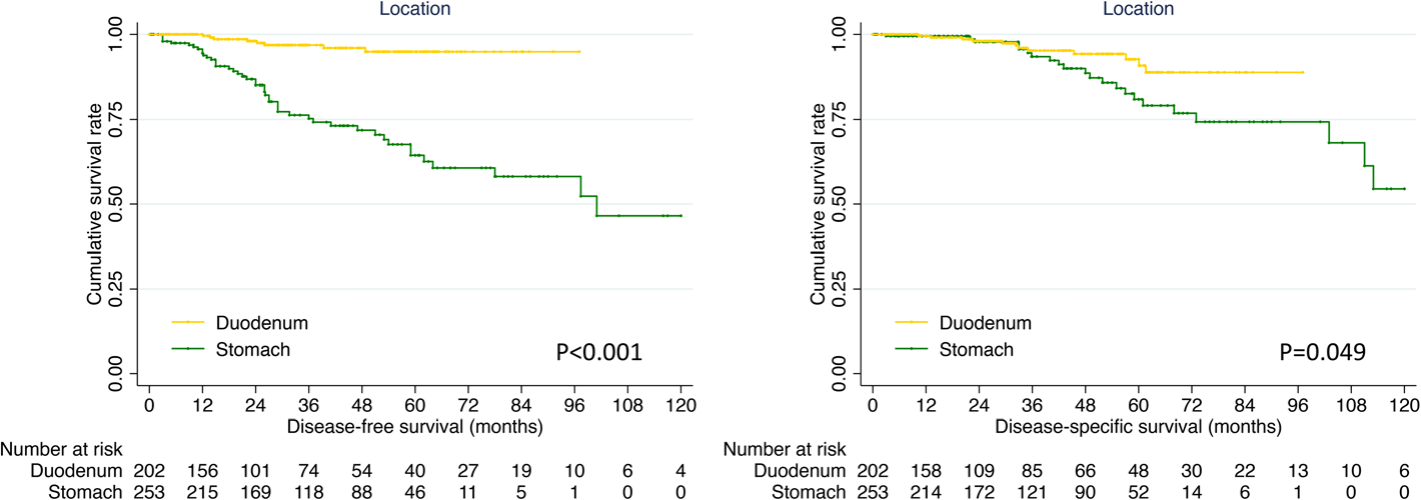
Comparison of DFS and DSS between duodenal and gastric GISTs

In order to analyze the prognosis of duodenal and gastric GISTs, survivals of 202 duodenal GISTs were compared to those of 253 gastric GISTs according to the exclusion criteria of survival analysis. The univariate and multivariate analysis showed that location was an independent risk factor for DFS and DSS (*P* < 0.001, Table 7). As shown in Fig. 4, the 5-year DFS (64.4% vs 94.9%, *P* < 0.001) and DSS (80.9% vs 92.6%, *P* = 0.049) of duodenal GISTs were significantly worse than that of gastric GISTs.

**Table 7:** Univariate and multivariate analysis of prognostic factors for duodenal and gastric GISTs

Prognostic factors	Univariate analysis			Multivariate analysis		
	β	Hazard ratio (95% CI)	*P* value	β	Hazard ratio (95% CI)	*P* value
DFS						
Age	−0.084	0.920 (0.517–1.638)	0.776			
Gender	−0.393	0.675 (0.384–1.186)	0.172			
Location	−2.105	0.122 (0.057–0.259)	0.000	−2.122	0.120 (0.054–0.266)	< 0.001
Tumor size	1.451	4.268 (2.932–6.213)	0.000	1.417	4.124 (2.526–6.733)	< 0.001
Mitotic index	1.283	3.608 (1.868–6.970)	0.000	0.928	2.528 (1.225–5.219)	0.012
NIH risk category	1.813	6.128 (3.254–11.544)	0.000			
DSS						
Age	0.387	1.473 (0.748–2.898)	0.263	0.759	2.136 (1.040–4.384)	0.039
Gender	0.279	1.322 (0.668–2.614)	0.423			
Location	−0.718	0.488 (0.236–1.010)	0.049	−1.066	0.344 (0.164–0.725)	0.005
Tumor size	1.297	3.658 (2.304–5.807)	0.000	1.386	3.999 (2.408–6.641)	< 0.001
Mitotic index	1.202	3.327 (1.574–7.032)	0.002			
NIH risk category	1.094	2.985 (1.821–4.895)	0.000			

GIST: gastrointestinal stromal tumor; NIH: National Institutes of Health;

DFS: disease-free survival; DSS: disease-specific survival; CI: confidence interval

## Discussion

The current study represented the largest number of duodenal GISTs to date. We found that LR was a more prevalent surgical procedure and PD was mainly performed for tumors with larger diameter or located in descending portion. Type of resection was not an independent risk factor for the prognosis of duodenal GISTs. Prognosis of duodenal GISTs was significantly worse than that of gastric GISTs.

GISTs are thought to derive from the interstitial cells of Cajal (ICC) [114], the pacemaker cells of gastrointestinal tract [115, 116]. A recent study found that the type of ICC distributed in proximal duodenum is very similar to that in stomach, and its distal duodenal pattern is more identical to that in jejunoileum [117]. Moreover, they found that ICC of circular muscle are only distributed in the proximal duodenum and are absent in the distal portion. In our study, most tumors located in the proximal portion of duodenum (superior and descending portion), which was consistent with the previous literature [1, 9, 13, 118]. This distribution characteristics may attribute to the distribution of ICC in this region. However, this remains to be further investigated.

Surgical strategy of duodenal GISTs remains challenging, owing to the unique anatomy of duodenum [91]. Complete surgical resection with sufficient margin and without intraoperative tumor rupture remains as the curative treatment for GISTs [2, 119]. Tumor size, location and invasion of adjacent organs are generally considered for the choice of surgery for duodenal GISTs [13, 120]. A few studies proponing PD as a routine procedure argued that an extensive surgery is always required in the pancreaticoduodenal region to obtain a clear margin and achieve a good oncological outcome [7, 13, 121]. On the other hand, LR, a less demanding procedure, could obviously decrease the perioperative morbidity and brings a parallel [121, 122] or better survival compared with PD [14]. A meta-analysis suggested LR as the routine choice for the duodenal GISTs whenever technically feasible, due to the good oncological outcomes and lower morbidity brought by this procedure compared with PD [118]. However, these results were all based on small samples. In our study, PD was mainly performed for GISTs with larger tumor size, higher risk-category or arose from descending portion. Although PD was associated with poorer survival of patients, surgical procedure was not an independent prognostic factor for duodenal GISTs. The survival disadvantage of PD observed in our study may be due to the higher-risk tumors distributed in the PD group.

In fact, the argument about LR and PD for duodenal GISTs mainly focused on tumors located in the descending portion. To date, study focused on this issue is lacking. In our study, PD was mainly performed for the descending GISTs. And, due to the particularly anatomic features of the duodenal descending portion, we then investigated the survival impact of surgical procedure for this subgroup of GISTs. The results showed that patients with descending GISTs underwent PD had larger tumor size and poorer DFS and DSS than those of patients underwent LR. However, multivariate analysis revealed that surgical procedure was not an independent prognostic factor.

Although our study indicated that type of resection was not associated with the prognosis of duodenal GISTs, the conclusion should be interpreted cautiously. For example, PD was the only choice to achieve a clearance margin when tumors were too large or close to the anatomically disadvantageous region. Thus, it is meaningless to compare the clinical impact of different types of resection without consideration of size and location of tumor. These two procedures could be compared only when the tumor is not large enough and is distant from the critical structures. However, to date, there is no more detailed study published. It is also a limitation in our study that the information of tumor location and involvement of the pancreaticoduodenal complex could not be extracted from published literatures.

Beside tumor size and mitotic index, tumor location is also reported as a key prognostic factor for GISTs [123, 124]. There are three main risk-stratification methods used to estimate the prognosis of GIST after surgery: NIH consensus criteria [125], Armed Forces Institute of Pathology (AFIP) criteria [126] and modified NIH criteria [113]. The latter two both include tumor site but only the AFIP criteria stratifies site into stomach, duodenum, jejunum and rectum while the modified NIH criteria only encompasses stomach and non-stomach. Even though, the comparison of survival between duodenal GISTs and GISTs from other sites was still rare due to the extremely low incidence [65]. Thus, we compared the prognosis of duodenal GISTs to gastric GISTs from our center. The univariate and multivariate analysis revealed that the DFS and DSS of duodenal were significantly worse than those of gastric GISTs. However, a recently nation-wide study [127] extracting GIST cases from Surveillance, Epidemiology, and End Results (SEER) database showed that gastric and small intestine GISTs had similar outcomes. This contrary result might because duodenal GIST was not analyzed separately from the small intestine GIST in their study which could lead to a bias. Actually, there is also a deficiency in current study, that the number of gastric GISTs in our study was relatively small compared to the large number of duodenal GISTs.

There are some other limitations in current study. Firstly, it is a retrospective single-center study and the completeness of systematic data is limited. Till now, the survival impact of surgical procedure on duodenal GISTs is still controversial, mainly because the lack of more accurate description of location of tumors in previous studies, which could result in a bias. Although the current study contained the largest number of duodenal GISTs, it still failed to make up this deficiency. Thus, a multi-center randomized control trial is needed to clarify this question. Secondly, due to the small size of small intestinal and colorectal GISTs in our center, the prognosis of duodenal GISTs were only compared to that of gastric GISTs.

## Conclusions

The most common symptom of duodenal GISTs was bleeding. Descending portion was the most frequent tumor site. LR was a more prevalent surgical procedure and PD was mainly performed for tumors with larger diameter or located in descending portion. But type of resection was not an independent risk factor for the prognosis of duodenal GISTs. Thus, the choice of surgical strategy of duodenal GISTs prevalently depended on tumor size and location. Prognosis of duodenal GISTs was significantly worse than that of gastric GISTs.

## Additional file

Additional file 1: **Table S1.** The comparison of clinicopathological features of duodenal GISTs between our center and published data. **Table S2.** The comparison of clinicopathological features of duodenal GISTs between published data and the entire cohort. **Figure S1.** The comparison of survival. We analyzed our own data (37 cases) and compared to the published combined data (263 cases). Then compared the 263 cases to the total combined 300 cases. The results showed that there was no significant difference in the results of the two comparisons. (DOCX 7148 kb)

## Abbreviations

DFS: Disease-free survival
DSS: Disease-specific survival
GISTs: Gastrointestinal stromal tumors
ICC: Interstitial Cajal cells
LR: Limited resection
PD: Pancreaticoduodenectomy
SEER: Surveillance, Epidemiology, and End Results
