# Supplementary material for: Clinicopathological features, surgical strategy and prognosis of duodenal gastrointestinal stromal tumors: a series of 300 patients
Source: BMC Cancer. 2018 May 15;18:563. doi: 10.1186/s12885-018-4485-4 (PMC5952823; doi:10.1186/s12885-018-4485-4)
Supplement: Supplementary file 1 — Table S1. The comparison of clinicopathological features of duodenal GISTs between our center and published data. Table S2. The comparison of clinicopathological features of duodenal GISTs between published data and the entire cohort. Figure S1. The comparison of survival. We analyzed our own data (37 cases) and compared to the published combined data (263 cases). Then compared the 263 cases to the total combined 300 cases. The results showed that there was no significant difference in the results of the two comparisons. (DOCX 7148 kb) [file 12885_2018_4485_MOESM1_ESM.docx]

We analyzed our own data (37 cases) and compared to the published combined data (263 cases). Then compared the 263 cases to the total combined 300 cases. The results showed that there was no significant difference in the results of the two comparisons.

**Table 1. The comparison of clinicopathological features of duodenal GISTs between our center and published data.**

|  | Our center (37) | Publications (263) | *P* value |
| --- | --- | --- | --- |
| Age |  |  | 0.005 |
| ≤60 | 30 (81.1%) | 131 (56.7%) |  |
| >60 | 7 (18.9%) | 100 (43.3%) |  |
| Gender |  |  | 0.508 |
| Male | 20 (54.1%) | 123 (48.2%) |  |
| Female | 17 (45.9%) | 132 (51.8%) |  |
| Anatomical location |  |  | 0.883 |
| Superior portion | 5 (14.3%) | 37 (15.9%) |  |
| Descending portion | 17 (48.6%) | 121 (52.2%) |  |
| Horizontal portion | 9 (25.7%) | 56 (24.1%) |  |
| Ascending portion | 4 (11.4%) | 18 (7.8%) |  |
| Surgery procedure |  |  | 0.936 |
| Limited resection | 22 (71.0%) | 177 (71.7%) |  |
| Pancreaticoduodenectomy | 9 (29.0%) | 70 (28.3%) |  |
| Tumor size |  |  | 0.256 |
| ≤2 cm | 2 (5.9%) | 32 (13.1%) |  |
| 2-5 cm | 21 (61.8%) | 114 (46.7%) |  |
| 5-10 cm | 9 (26.5%) | 65 (26.6%) |  |
| >10 cm | 2 (5.9%) | 33 (13.5%) |  |
| Mitotic index |  |  | 0.382 |
| ≤5 | 20 (69.0%) | 162 (76.4%) |  |
| >5 | 9 (31.0%) | 50 (23.6%) |  |
| Morphology |  |  | 0.165 |
| Spindle | 35 (100%) | 114 (90.5%) |  |
| Epithelioid | 0 | 1 (0.8%) |  |
| Mixed | 0 | 11 (8.7%) |  |
| NIH risk category |  |  | 0.420 |
| Very low | 1 (3.1%) | 24 (10.6%) |  |
| Low | 16 (50.0%) | 88 (38.8%) |  |
| Intermediate | 0 | 2 (0.9%) |  |
| High | 15 (46.9%) | 113 (49.8%) |  |

**Table 2. The comparison of clinicopathological features of duodenal GISTs between published data and the entire cohort.**

|  | Publications (263) | Entire cohort (300) | *P* value |
| --- | --- | --- | --- |
| Age |  |  | 0.417 |
| ≤60 | 131 (56.7%) | 161 (60.3%) |  |
| >60 | 100 (43.3%) | 106 (39.7%) |  |
| Gender |  |  | 0.833 |
| Male | 123 (48.2%) | 143 (49.1%) |  |
| Female | 132 (51.8%) | 148 (50.8%) |  |
| Anatomical location |  |  | 0.996 |
| Superior portion | 37 (15.9%) | 42 (15.8%) |  |
| Descending portion | 121 (52.2%) | 137 (51.5%) |  |
| Horizontal portion | 56 (24.1%) | 65 (24.4%) |  |
| Ascending portion | 18 (7.8%) | 22 (8.3%) |  |
| Surgery procedure |  |  | 0.963 |
| Limited resection | 177 (71.7%) | 199 (71.8%) |  |
| Pancreaticoduodenectomy | 70 (28.3%) | 78 (28.2%) |  |
| Tumor size |  |  | 0.966 |
| ≤2 cm | 32 (13.1%) | 34 (12.3%) |  |
| 2-5 cm | 114 (46.7%) | 135 (48.7%) |  |
| 5-10 cm | 65 (26.6%) | 73 (26.4%) |  |
| >10 cm | 33 (13.5%) | 35 (12.6%) |  |
| Mitotic index |  |  | 0.804 |
| ≤5 | 162 (76.4%) | 181 (75.4%) |  |
| >5 | 50 (23.6%) | 59 (24.6%) |  |
| Morphology |  |  | 0.829 |
| Spindle | 114 (90.5%) | 148 (92.5%) |  |
| Epithelioid | 1 (0.8%) | 1 (0.6%) |  |
| Mixed | 11 (8.7%) | 11 (6.9%) |  |
| NIH risk category |  |  | 0.979 |
| Very low | 24 (10.6%) | 25 (9.7%) |  |
| Low | 88 (38.8%) | 104 (40.3%) |  |
| Intermediate | 2 (0.9%) | 2 (0.8%) |  |
| High | 113 (49.8%) | 127 (49.2%) |  |


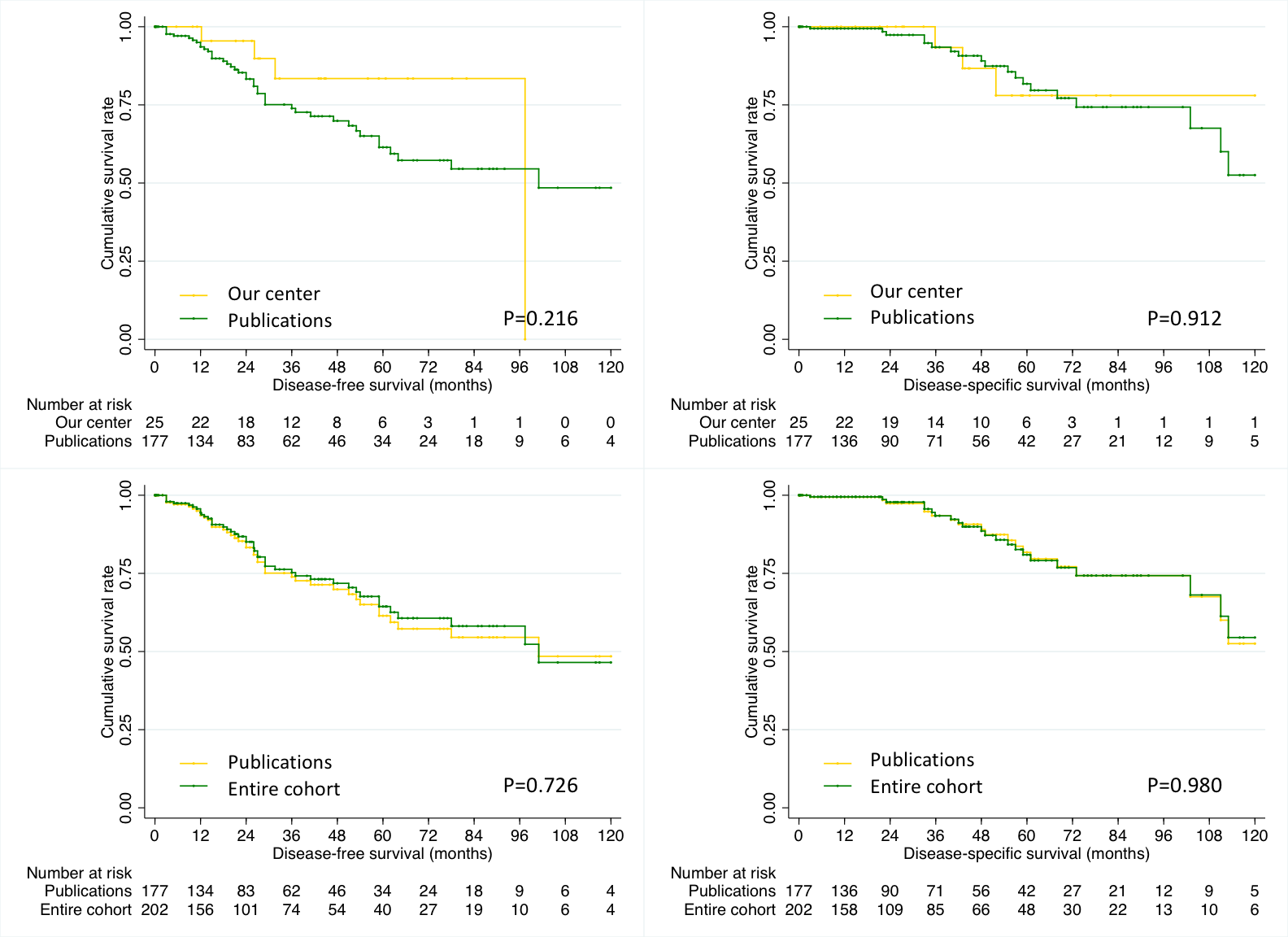


**Figure 1. The comparison of survival.**
